# Global Is Local: Interprofessional Experiential Learning for Migrant Farmworker Health Equity

**DOI:** 10.1089/heq.2021.0114

**Published:** 2022-03-03

**Authors:** Benjamin C. Katz, Emily P. Syverud, Oscar W. Garza, Rachel Silva, Jonathan D. Kirsch

**Affiliations:** ^1^ Department of Pediatrics, University of Minnesota Medical School, Minneapolis, Minnesota, USA.; ^2^ Department of Internal Medicine, University of Minnesota Medical School, Minneapolis, Minnesota, USA.; ^3^ College of Pharmacy, University of Minnesota, Minneapolis, Minnesota, USA.; ^4^ Department of Internal Medicine, Hennepin Health Care, Minneapolis, Minnesota, USA.

**Keywords:** migrant health, farmworker, health equity, medical education, interprofessional education

## Abstract

**Purpose:**

Medical educators recognize the importance of addressing social determinants of health and providing opportunities for learners to work with diverse populations. Still, social, geographic, resource, and language barriers prevent institutions from connecting with globally diverse populations within their own communities. In this article, we describe the migrant farmworker health course at the University of Minnesota, the importance of longitudinal partnership with community-based organizations, and ways to increase access to care and educate health professional learners in health equity.

**Methods:**

The migrant farmworker health course is a clinical rotation that combines didactic learning on social determinants of health with hands-on clinical experience. Learners work with community organizations to provide mobile health care while learning about a diverse and underserved rural population. Twenty-eight learners who participated in the course between 2015 and 2019 were surveyed about their experience, knowledge, and skills gained, and recommendations for improvement.

**Results:**

Over 90% of participants rated their overall experience in the course as “good” or “outstanding.” Most learners increased confidence in the subjects that were covered during didactic sessions. Qualitative feedback provided insight on how the migrant farmworker health course shaped learners' understanding of social determinants of health and influenced their career trajectories.

**Conclusion:**

The migrant farmworker health course has educated interprofessional learners and is expanding to include more opportunities for mobile health care. The feedback from this survey helped improve didactic teaching and will help deepen relationships with community partners. Learning through service with global populations locally in a “global is local” rotation is a rewarding way to engage in and learn about health equity.

## Introduction

Current medical education institutions struggle in training future health care providers to address inequities affecting those of disempowered populations whether it be due to their race, ethnicity, class, or geographic location, particularly when these marginalizations intersect.^1^ Despite the recent push for interprofessional education, health education remains remarkably siloed by profession, with limited interactions between students preparing for health care and ancillary professional careers.^2^ Furthermore, although many major academic medical centers have programs for learners to travel abroad to work with underserved populations, they have limited opportunities to serve and learn from globally diverse and underserved populations within their own communities.^3^

More than one-quarter of medical students have had medical rotations abroad by the time they enter residency,^4^ and an increasing number of residency programs have formal training opportunities through global health pathways and certificate programs.^4^ Residents who choose this option during their training generally go abroad to work with medically underserved and impoverished populations.

However, there is limited reporting in the medical literature about curricula and training opportunities to work with globally diverse rurally based domestic populations outside of traditional urban medical structures such as hospital-based clinics, free student-run community clinics, and residency-based community health centers. The literature suggests that such unique opportunities have an important impact on learners' exposure to and confidence in treating diverse populations.^5^

In Minnesota, ∼30,000 migrant farmworkers travel annually to rural regions of the state for short-term agricultural work. They experience multiple barriers to health care access including language (most are primarily Spanish speaking), limited healthy food access, substandard living conditions, and limited to no access to health insurance.^6,7^ Moreover, migrant farmworkers work long shifts and lack transportation, making visits to medical clinics difficult.^6,8^ This population also faces unique occupational hazards including overuse injury, machinery accidents, and pesticide exposures from hours of labor in the fields.^7^

Not only do migrant farmworkers face several significant barriers to accessing any health care, but also these systems and providers are not equipped to sufficiently address the multifaceted needs of these essential workers and their families.^9^ Although academic institutions are attempting to integrate social determinants of health and racial and geographic inequities into their curricula, health care delivery systems often find themselves lacking the financial, personnel, and community resources to effectively address these same issues.^10^

The migrant farmworker health course at the University of Minnesota fills this gap by educating learners from various health disciplines about the factors affecting migrant farmworker community health outcomes while concurrently partnering with public and community programs to provide health services to these communities.

This unique university educational experience was developed by partnering with public programs, advocacy groups, and community-based organizations. Comprehensive background instruction combined with service opportunities allowed students to apply classroom knowledge in patient care, particularly when the classroom is set in a farm, dairy, cannery, consulate, rural public health department, and a migrant farmworker housing camp.

Through this interprofessional experiential learning opportunity to work with a globally diverse population in rural Minnesota, students are able to understand the complex global and local layers of health inequities and how they intersect. Through creative community partnerships, the course successfully inspired and trained future generations of health care workers to address these local inequities by showing them how to cross the traditional borders erected by a clinic's physical walls.

## Methods

### Course design

We developed a 4-week interprofessional community-engaged mobile health rotation for resident physicians, medical students, dental students, pharmacy students, and undergraduate students. This rotation was the result of collaboration by faculty in the University of Minnesota Medical School, College of Pharmacy and School of Dentistry. The Department of Chicano and Latino Studies was consulted in the initial course design, as was a community organizing nonprofit that has advocated for migrant farmworkers in Minnesota for decades. Clinical care was provided by university faculty in partnership with local community organizations and federally qualified health centers that provide care to farmworkers.

The primary aim of the course was twofold. First, we sought to provide Minnesota's migrant and seasonal farmworkers and their families with faculty-supervised medical screenings, assessments, and treatment. Second, through didactics, we strived to educate the students regarding specific social determinants of health affecting migrant and seasonal farmworkers in Minnesota and the rest of the United States.

### Course implementation

Before course initiation, participants completed an online “Clinician Orientation to Migrant Health,” a series of seven webinars, to familiarize themselves with farmworker health in the United States.^11^ All participants received training on working with interpreters and non-English-speaking patients before the course. Early didactic sessions included overviews of the history of farm labor and immigration in the United States, workers' compensation and its variations across state lines, occupational health, community health workers, and environmental health. Interactive sessions regarding the multifaceted forms of health care access and how to approach health care through the lens of human rights theory also took place during the course's initial days.

Our primary clinical partner was the Tri-Valley Opportunity Council (TVOC), which operates the Migrant and Seasonal Head Start (MSHS) programs in Minnesota for children of farmworkers. Several of their staff have been working with the same migrant farmworkers for years and even were once farmworkers themselves. Their pre-established ties and community trust were essential to any health outreach efforts by our course participants. Children in their MSHS programs are required to have screening physical, vision, hearing, and oral health examinations.

Under the supervision of Spanish- and English-speaking faculty board certified in internal medicine, pediatrics, and family medicine, our medical learners performed these screening histories and examinations. The TVOC staff contributed support and guidance to learners and coordinated any follow-up care and appointments. TVOC also cohosted course organized health fairs to engage workers and families, educate about relevant community resources, and provide medical and dental evaluations to migrant farmworkers and their families.

Interspersed with clinical sessions, experiential learning and didactic training sessions educated learners regarding the complex social determinants of health affecting migrant and seasonal farmworkers and their families (Table 1). Students visited local farms, canneries, and dairies to observe these work environments and further grasp the occupational health risks of agricultural work. We were invited to visit the families' temporary employer-provided housing that provided context for students to incorporate when providing care. We also met with the local Mexican consulate, county health department offices, and free clinics as part of the course's asset mapping objective to navigate the patchwork of resources available to this patient population and the challenges these organizations face to provide care.

**Table 1.** Demographics of Course Participants

Demographic	*n* (%)
Participant year (*N*=28)	
2015	7 (25.5)
2016	1 (3.6)
2017	3 (10.7)
2018	9 (32.1)
2019	8 (28.6)
Level of training (*N*=28)	
Medical resident	14 (50.0)
Medical student	6 (21.4)
Dental student	2 (7.1)
Other (pharmacy, undergraduate, etc.)	6 (21.4)
Gender identity (*N*=22)	
Male/man	5 (17.9)
Female/woman	17 (60.7)
Self-identified race/ethnicity (*N*=21)	
White (non-Hispanic)	13 (46.4)
White (Hispanic)	1 (3.6)
Hispanic/Latinx	5 (17.9)
Asian	1 (3.6)
Black	1 (3.6)
Age (*N*=21)	
≤30	14 (50.0)
≥31	7 (25.5)

Learners visited local advocacy groups such as Centro Campesino to learn the history of labor and community organizing in rural Minnesota and the struggles currently facing these communities. An annual guest lecture by a staff lawyer with Southern Minnesota Regional Legal Services educated students about the injustices that farmworkers face ranging from wage theft, substandard housing, unsafe working conditions, and other labor violations. The organization served as a referral opportunity for patients who required free high-quality legal assistance and educating students about the importance of medical–legal partnerships.

Learners also had the opportunity to learn with youth from local migrant and seasonal farmworker communities about social determinants of health as well as provide informal mentorship and discuss the pathways to health professional education.

Other didactic sessions, including lectures, films, and readings, provided further background knowledge for the learners. Lectures were largely interactive and discussion based. Lecturers were chosen for their content expertise, including national leaders and researchers when possible. Content was broad and included pesticide risk and toxicity management, farm work ergonomics, food systems, agricultural medicine, occupational dermatology, immigration law, labor law, oral health, nutrition education, women's health, and narrative medicine.

Guest lecturers included activists advocating for environmental health improvements, occupational medicine physicians, global migration health experts, environmental health experts, local chefs and local internal medicine, pediatrics, and family medicine physicians who work with Latinx and immigrant communities. The course also assigned learners to choose a topic of interest and lead journal club discussions of articles relevant to the course.

### Course survey

We performed a literature review through PubMed to examine how others have surveyed learners following elective courses, specifically those who involved a global health or rural health component. We used this background to design a survey that assessed participant demographics, overall experience in the course, and the knowledge and experience that participants gained. The survey included Likert-type questions and free-response items. The survey was shared with all past participants electronically through REDCap (Research Electronic Data Capture), a secure web-based software platform hosted at the University of Minnesota.^12^ Completion was voluntary and uncompensated. The University of Minnesota Institutional Review Board approved the survey (STUDY00007243).

## Results

### Participant characteristics

Thirty-three learners, mostly from various academic health center schools and residency programs at the University of Minnesota, have participated in the migrant farmworker health course since its inception in 2015. Twenty-eight participants responded to our survey and provided both quantitative and qualitative feedback about the program. Table 2 outlines the demographics of the participants.

**Table 2.** Grading of Experience

Categories and items	Median	Median absolute deviation
Course experience/impact		
Overall experience	4	0
Felt like a “global health rotation”	3	0
Course altered your career trajectory	3	1
Recommend the course to…		
All students/residents	4	0
Students/residents that speak Spanish	4	0
Students/residents interested in Global Health	4	0
Students/residents interested in rural health	4	0

Rating scale: strongly disagree (1), disagree (2), agree (3), strongly agree (4).

The majority (71.4%) were either medical students or medical residents. Others who participated included dental students, high school students, and undergraduate students. Five participants (17.9%) identified as Latinx. The majority (60.7%) identified as female. Three participants (10.7%) were born outside of the United States. One participant from 2015 did not respond because he became an instructor of the course from 2017 to 2019.

### Overall experience

As part of our survey, we wanted to understand the participants' rating of the course overall, through both quantitative and qualitative feedback. When asked about their general experience in the course, >90% of participants rated it as “good” or “outstanding” (Table 3). We also found that most participants (60.7%) would recommend this course to any resident or student and an even higher percentage would recommend it to those interested in global health (78.6%) or rural health (88.5%; Table 3). Single factor analyses of variance (ANOVA) did not demonstrate any significant differences in grading of experience between groups based on participant year or participant's school of origin.

**Table 3.** Knowledge Gains

Categories and items	Median	Median absolute deviation
Increased confidence and understanding of…		
Working with community partners	3	0.5
Providing interprofessional health care in a rural setting	3	0
Increased confidence in ability to…		
Interact with people of diverse backgrounds	4	1
Explain the difference between a stereotype and an assumption	4	0
Identify the influence of stereotypes on your thoughts, feelings, and behavior	4	1
Elicit a patient's perspective of healing	4	1
Effectively use a skilled interpreter	4	1
Increased understanding of social determinants of health for…		
Migrant agricultural workers	4	0
Rural Minnesotans	4	0
Latinx patients	4	0
Spanish-speaking patients	4	0
Increased knowledge of…		
Worker's compensation	3	1
Immigration law	3	0
Labor law	3	1
Public health resources	4	0
Health risks of field farm work	4	0
Health risks of factory work	4	0
Health risks of dairy work	4	0
Working with community health centers	4	0
Mobile health	3	1
Health care in low-resource settings	3.5	0.5
Health and human rights	4	0
Environmental health	3	0
Child labor	3	0
Narrative medicine	3	1
Advocacy for vulnerable populations	4	0
Food systems	3	1
Nutrition	3	1
Pesticides and health	4	0.5

Rating scale: not at all (1), a little (2), somewhat (3), a significant amount (4).

In a separate section, we received qualitative feedback on the most and least valuable experiences and general recommendations for improvement. These data demonstrated that many participants enjoyed opportunities to experience first hand the occupational environments of migrant farmworkers across a variety of workplaces, such as canneries and dairy farms, and gained a better understanding of the lives of the patients they saw in the clinics. As one participant wrote, “I never would have learned about the disparities and specific health needs of this population if it had not been for this elective.”

Our hypothesis about the lack of exposure to local health inequities was confirmed by participant feedback and provided a widened lens for many participants hoping to affect change within their own communities of practice. We also identified areas for improvement, including reorganizing the presentation of specific didactic lectures and clarifying general course organization and communication.

### Knowledge gains

The migrant farmworker health course uses both clinical experience and didactic sessions to educate participants about topics that are not covered in many traditional curricula. The survey assessed how well the course educated students about these topics, including social determinants of health, stereotypes, health risks specific to migrant farmworkers, and more.

Table 4 outlines participants' median responses to questions about the knowledge, understandings, and abilities they gained through participation in the course. On average, responses indicated that students felt “moderately confident” or “very confident” about most topics that were covered.

**Table 4.** Migrant Health Course Educational Domain Curriculum

Didactic	Experiential	Clinical
History of farm labor	Vegetable processing factory tour	Migrant education program screening physicals
Social determinants of health overview	County public health department tour	Interprofessional multiagency health fairs
Environmental health and pesticides	Rural community health center tour	Free clinic of Steele County
Community health workers	“Day on the farm” with Manos Latinas Cooperative	
Working with interpreters	Migrant farmworker housing camp visit	
Occupational health and workers' compensation overview	Dairy tour and discussion with dairy workers and owner	
Overview of immigration law and know your rights training	Mexican consulate visit	
One Health—the connection between animal, human, and ecological health	Centro Campesino visit: community organizing in Minnesota	
Southern Minnesota Regional Legal Services—overview of labor legal issues affecting migrant farmworkers	Supplementary videos: - *Los Lecheros* - *Harvest of Shame* - *Food Chains* - *Abrazos*	
Food systems and how to discuss nutrition with patients		

We also assessed the course's efficacy in achieving educational goals outside the classroom, such as interacting with people from diverse backgrounds and working on an interprofessional team. Most students “agree” (60.7%) or “strongly agree” (21.4%) that the course increased their confidence and understanding of providing interprofessional health care (Table 3). The qualitative feedback encouraged us to implement more interprofessional collaboration to provide the best care for migrant and seasonal farmworkers and their families. Didactic, experiential, and clinical domains are highlighted in Table 4 and a specific curriculum is included as a Supplementary Table S1.

The feedback regarding collaboration with community partners was overwhelmingly positive, with many participants commenting on how the strong relationship with community partners helped the relatively short elective course build trust with the community year after year.

## Discussion

The migrant farmworker health course effectively educated its learners on the complicated social determinants of health affecting this population and how to integrate this knowledge into direct clinical care. A learner highlighted that the depth of the “course's didactic background in the history of migrant labor in the US was some of the most robust exposure I have had to these issues.” The experiential learning from didactics and direct clinical care was powerful as “it was really valuable to have read and heard a lot about the lives of migrant farmworkers through lectures and then work with migrant farmworker individuals and families to get a more concrete picture of their experiences.” This feedback reinforced the importance of addressing these specific subject matters that are often neglected in traditional medical curricula.

The unique interprofessional nature and experiential learning aspects of the course also provided learners with important lessons. One student described how “having a holistic team sharing their experiences in their education allowed me to better identify gaps in how healthcare is provided thus impacting how I currently approach it with patients that I serve.” An aspect that participants found particularly valuable was the experience of asset mapping “all of the resources that some of these very rural communities have for their migrant farmworker populations.”

The various site visits allowed learners to comprehend the broader patient experience. Bringing students outside of the traditional classroom proved to be an impactful educational method to provide them with more tools to serve not only migrant farmworkers but also patients of Latinx and rural backgrounds.

The course also successfully educated learners of not only the importance of community partnerships but also how to build and nurture them. As one participant described “it was always communicated the importance of building trust and maintaining relationships in order to assist one another in achieving the same goals working with the migrant farmworkers and their families.” They also recognized “how working with community partners can open up huge opportunities for both the community and for the healthcare team” and “how healthcare can be adapted to meet local needs in underserved communities in flexible and culturally responsive manners.”

The migrant farmworker health course addresses an unserved need both for our patients and our learners. Through meaningful partnership with community health organizations, we are improving access to care for an extremely marginalized group. At the same time, we are providing an opportunity for students to work with an interprofessional team and learn about social determinants of health in both didactic and clinical settings. Many participants provided meaningful feedback about how the course has altered the trajectory of their career and encouraged them to continue working with the organizations and individuals they met through the course.

The feedback in this course suggests that students need not travel far from their home institutions for training to work with globally diverse and medically underserved populations. They also learn the importance of building a trusting relationship with community partners, just as their local institutions partner with organizations overseas. Given the vast number of farmworker populations across rural United States, this course can serve as a model for similar type clinical partnerships and learning opportunities for often urban-based resourced health care training institutions, particularly for students interested in working with globally diverse and underserved patient populations.

The evaluation of the course gave us a clear direction for improvement. The feedback regarding didactic lectures has guided to further clarify learning outcomes for each session and standardize preparation and delivery across topics. We also have and will continue to increase opportunities for interprofessional care by partnering further with academic health center schools outside of the medical school. The data from survey participants will provide us with compelling evidence to seek further institutional support for our course to maintain a cross-cultural educational experience.

We also aim to further our partnerships with TVOC, rural health providers, and other training programs to expand our faculty and student pool. As of the time of this writing and based on learner feedback, we have secured funding for a new mobile medical unit that will better serve our learners and patients year-round. We will continue to use the same survey in future years to track participant responses to various changes in the course.

### Limitations

Participants were self-selected and likely to be interested in social determinants of health, global health, and/or rural health and thus may be more likely to feel that this was relevant to themselves and others. Owing to the small number of participants, it would be difficult to show a statistically significant difference in the career trajectory, though anecdotally many participants expressed interest in and have incorporated mobile, rural, or Latinx health into their careers.

## Conclusion and Health Equity Implications

By combining relevant in-depth social determinants of health education, engaging community partnerships, and clinical training opportunities, there are numerous opportunities to equip the next generation of health care workers to not only provide care for migrant farmworkers but also advocate for them. Students can learn of their ability to impact health outcomes in populations that are geographically, socioeconomically, and racially marginalized, particularly when they intersect.

Furthermore, practice in all health fields is increasingly interprofessional and learners need experience to understand how to communicate across disciplines. Health equity requires active efforts by health professional training programs to provide opportunities to learn and serve, by investing in longitudinal community-based partnerships. Learners interested in global health should seek out opportunities to work with global populations locally and ask their institutions to provide “global is local” rotations.

## Supplementary Material

Supplemental data

## Abbreviations Used

MSHS: Migrant and Seasonal Head Start
TVOC: Tri-Valley Opportunity Council
