# Supplementary material for: Global Is Local: Interprofessional Experiential Learning for Migrant Farmworker Health Equity
Source: Health Equity. 2022 Mar 3;6(1):159–66. doi: 10.1089/heq.2021.0114 (PMC8896175; doi:10.1089/heq.2021.0114)
Supplement: Supplemental data [file Suppl_TableS1.docx]

**Supplement: Migrant Health Elective 2019 Calendar**

| Monday | Tuesday | Wednesday | Thursday | Friday |
| --- | --- | --- | --- | --- |
| July 2  Orientation & Syllabus  Ground Rules  Making a Healthcare System Brainstorm  Projects and care teams  History of farm labor  Harvest of Shame Debrief | July 3  Orientation with CBO  Farm Visit  PM Journal club  Social determinants of health  Environmental Health and Pesticides | July 4:  Break for Independence Day | July 5  AM Journal Club  Health Promoters / community health workers  Working with Interpreters  Dairy farmworkers film: Los Lecheros | July 6  Self study  Occupational health and zoonotic infections  Legal aid for migrants, know your rights training |
| July 9  Clinical: physicals for the Migrant Education Program  County Public Health Tour | July 10  Clinical: physicals for the Migrant Education Program  Vegetable processing factory tour | July 11  Clinical: physicals for the Migrant Education Program | July 12  Evening Farmworker Parent Meeting and Health Family night | July 13  Role of the Mexican Consulate  Journal club |
| July 16  Community health center tour  Farmworker Youth meeting with pipeline students | July 17  Clinical: Physicals for the Migrant Education Program | July 18  Clinical: physicals for the Migrant Education Program | July 19  Clinical: physicals for the Migrant Education Program | July 20  One Health- the connection between animal, human and ecological health  Journal club |
| July 23  Clinical: physicals for the Migrant Education Program | July 24  Clinical: physicals for the Migrant Education Program | July 25  Journal club  Self-directed study, writing, poster preparation | July 26  Evaluations  Debrief with CBO  Evening clinic | July 27  Last day of rotation  Rotation breakfast for reflection at Latino food court |
